# Angiotensin II Type 1 Receptor-Dependent GLP-1 and PYY Secretion in Mice and Humans

**DOI:** 10.1210/en.2016-1384

**Published:** 2016-07-22

**Authors:** Ramona Pais, Juraj Rievaj, Pierre Larraufie, Fiona Gribble, Frank Reimann

**Affiliations:** Wellcome Trust-Medical Research Council Institute of Metabolic Science, Metabolic Research Laboratories, University of Cambridge, Addenbrooke's Hospital, Cambridge CB2 0QQ, United Kingdom

## Abstract

Angiotensin II (Ang II) is the key hormone mediator of the renin angiotensin system, which regulates blood pressure and fluid and electrolyte balance in the body. Here we report that in the colonic epithelium, the Ang II type 1 receptor is highly and exclusively expressed in enteroendocrine L cells, which produce the gut hormones glucagon-like peptide-1 and peptide YY (PYY). Ang II stimulated glucagon-like peptide-1 and PYY release from primary cultures of mouse and human colon, which was antagonized by the specific Ang II type 1 receptor blocker candesartan. Ang II raised intracellular calcium levels in L cells in primary cultures, recorded by live-cell imaging of L cells specifically expressing the fluorescent calcium sensor GCaMP3. In Ussing chamber recordings, Ang II reduced short circuit currents in mouse distal colon preparations, which was antagonized by candesartan or a specific neuropeptide Y1 receptor inhibitor but insensitive to amiloride. We conclude that Ang II stimulates PYY secretion, in turn inhibiting epithelial anion fluxes, thereby reducing net fluid secretion into the colonic lumen. Our findings highlight an important role of colonic L cells in whole-body fluid homeostasis by controlling water loss through the intestine.

The prime functions of the gut are the digestion and absorption of ingested food. These are regulated by intestinal hormones, such as glucagon-like peptide-1 (GLP-1) and peptide YY (PYY), which are cosecreted from enteroendocrine L cells found predominantly in the ileum and colon (1). Both hormones underlie the ileal break, slowing gastric emptying when nutrient delivery exceeds the absorptive capacity of the duodenum/jejunum, and control food intake and appetite (2). These effects beyond the confines of the intestine have raised interest in the exploitation of gut hormones for the treatment of diabetes and obesity. GLP-1 augments glucose induced insulin secretion (3) and has been exploited in the form of GLP-1 mimetics for the treatment of diabetes and obesity.

An additional action of PYY is to inhibit intestinal water and anion secretion. This is achieved through a direct action on enterocyte Y1 receptors and an indirect effect on Y2 receptors located on enteric neurons (4). This paracrine effect of PYY is important for body fluid and electrolyte homeostasis. We showed previously that arginine vasopressin (AVP) stimulates GLP-1 and PYY release from mouse and human colonic L cells and suggested that this forms part of a mechanism that reduces water loss through the intestine (5). Another important regulator of water and electrolyte balance and blood pressure (BP) is the renin angiotensin system (RAS) (reviewed in reference 6), which exhibits both systemic and local regulation. Sympathetic stimulation, renal artery hypotension, or reduced blood volume (eg, dehydration or hemorrhage) initiate the release of renin from renal juxtaglomerular cells, which converts circulating angiotensinogen to angiotensin I. Angiotensin I in turn is hydrolyzed by angiotensin-converting enzyme (ACE) to form the biologically active octapeptide angiotensin II (Ang II). Ang II causes arterial vasoconstriction and renal retention of sodium and fluid, and stimulates the release of aldosterone and AVP from the adrenal cortex and posterior pituitary, respectively.

Several studies have identified different components of the RAS, including angiotensinogen, renin, ACE, Ang II, and angiotensin receptors in the mucosal and muscular layers of the gastrointestinal tract (7–10). Angiotensin receptors, particularly Ang II type 1 (AT_1_), have been implicated in gut motility (11, 12) and electrolyte absorption (13–15). Here we report that AT_1_ is highly and selectively expressed in colonic L cells and is linked to the stimulation of PYY and GLP-1 secretion and colonic fluid balance.

## Materials and Methods

### Solutions and compounds

All compounds were purchased from Sigma-Aldrich unless otherwise stated. BIBP 32267 trifluoroacetate was purchased from Bioquote and angiotensin (1–7) from Bio-Techne. The composition of the standard bath solution used in the secretion and imaging experiments was as follows: 4.5 mmol/L KCl, 138 mmol/L NaCl, 4.2 mmol/L NaHCO_3_, 1.2 mmol/L NaH_2_PO_4_, 2.6 mmol/L CaCl_2_, 1.2 mmol/L MgCl_2_, and 10 mmol/L HEPES (adjusted to pH 7.4 with NaOH). For experiments in which CoCl_2_ was used, carbonates and phosphates were omitted from the saline buffer and the osmolarity was compensated with additional NaCl (143 mmol/L total). The composition of Ringer's solution used in Ussing chamber experiments was 120 mmol/L NaCl, 3 mmol/L KCl, 0.5 mmol/L MgCl_2_, 1.25 mmol/L CaCl_2_, 23 mmol/L NaHCO_3_, and 10 mmol/L glucose.

### Animals and ethical approval

All animal procedures were approved by the University of Cambridge Animal Welfare and Ethical Review Body and conformed to the Animals (Scientific Procedures) Act 1986 Amendment Regulations (SI 2012/3039). The work was performed under the UK Home Office Project License 70/7824. Male and female mice, aged 3–6 months, on a C57BL6 background were housed in individually ventilated cages on a 12-hour dark, 12-hour light cycle with ad libitum access to water and chow. Mice were euthanized by cervical dislocation and intestinal tissue used in the experiments. For in vivo experiments, only male mice, aged 11–12 weeks, were used. Mice were fasted overnight for a maximum of 16 hours before receiving an ip injection of either Ang II (100 μg/kg) or PBS (vehicle). Ten minutes after the injection, each animal was anesthetized (isoflurane) and a terminal blood sample taken. Blood was collected in tubes containing EDTA and protease inhibitors (10 μmol/L amastatin hydrochloride, 100 μmol/L diprotinin A, 18 μmol/L aprotinin), centrifuged at 13 000 × *g* for 90 seconds and plasma collected and used for active GLP-1 and total PYY analysis.

### Transgenic mice

GLU-Venus and GLU-Cre mice have been previously described (16, 17) and express the fluorescent protein Venus and *Cre* recombinase under the control of the proglucagon promoter, respectively. To monitor the calcium fluctuations in L cells, GLU-Cre mice were crossed with ROSA26-GCaMP3 reporter mice (18) (Jax stock 014538) to generate L cell-specific expression of the genetically encoded Ca^2+^ sensor.

### Primary murine colonic crypt cultures

Colonic crypts were isolated and cultured as previously described (16). Briefly, mice 3–6 months old were killed by cervical dislocation and the colon was excised. Luminal contents were flushed thoroughly with PBS and the outer muscle layer removed. Tissue was minced and digested with collagenase type XI (0.4 mg/mL) and the cell suspension plated onto Matrigel (BD Bioscience) precoated 24-well plates for GLP-1 secretion experiments or on 35-mm glass bottomed dishes (Mattek Corp) for live cell calcium imaging.

### Preparation of crypt cultures from human colons

The study was approved by the Research Ethics Committee under license number 09/H0308/24. Fresh surgical specimens of human colon were obtained from Tissue Bank at Addenbrooke's Hospital (Cambridge, United Kingdom), stored at 4°C, and processed within a few hours of surgery. The crypt isolation procedure was similar to that used for mouse tissue with the exception that a higher concentration of collagenase XI (0.5 mg/mL) was used for digestion (1).

### GLP-1 and PYY secretion assays

Eighteen to 24 hours after plating, cells were washed and incubated with test agents dissolved in standard bath solution supplemented with 0.1% BSA for 2 hours at 37°C. At the end of the incubation, supernatants were collected and centrifuged at 2000 rcf for 5 minutes and snap frozen on dry ice. Cells were lysed with lysis buffer containing 50 mmol/L Tris-HCl, 150 mmol/L NaCl, 1% IGEPAL-CA 630 (Sigma-Aldrich), 0.5% deoxycholic acid, and complete EDTA-free protease inhibitor cocktail (Roche) to extract intracellular peptides, centrifuged at 10 000 rcf for 10 minutes and snap frozen. GLP-1 and PYY were measured using total GLP-1 and total PYY assays (MesoScale Discovery), and supernatant concentrations were expressed as a percentage of the total (secreted+lysate) GLP-1 or PYY content of each well.

### Calcium imaging

L cell cytosolic calcium concentrations were monitored as intensity changes in GCaMP3 fluorescence excited at 488 nm using a xenon arc lamp and a monochromator (Cairn Research) in colonic crypt cultures prepared from GLU-Cre/ROSA26-GCaMP3 mice. Solutions were perfused continuously at a rate of approximately 1 mL/min. Imaging was performed using an Olympus IX71 microscope with a ×40 oil immersion objective and an OrcaER camera (Hamamatsu). Images were acquired at 1 Hz and analyzed, after background subtraction, using MetaFluor software (Molecular Devices). Fluorescence in the presence of the test agent was normalized to the respective mean background fluorescence of each cell, measured before the addition and after the washout of the test compound. For presentation, data were smoothened with a sliding average over 20 seconds.

### Microarray analysis and RNA sequencing

Microarray analysis of total RNA from fluorescence-activated cell sorter (FACS)-purified L cells using Affymetrix mouse 430 2.0 expression arrays (Affymetrix UK Ltd) has been described previously (19). Expression levels of each probe were determined by robust multichip average analysis. For sequencing, total RNA from 2000 to 10 000 FACS-purified L cells from the upper small intestine (top 10 cm), lower small intestine (bottom 10 cm), or colon/rectum from GLU-Venus mice was extracted using an RNeasy Micro Plus kit (QIAGEN) according to the manufacturer's instructions. RNA was amplified using the Ovation RNA-seq System V2 (NuGEN), using 1 ng of RNA for each sample (three replicates each were used for L cells and nonfluorescent control cells for each segment of the gastrointestinal tract, totaling 18 samples). To prepare the RNA sequencing library, the amplified cDNA (1 μg per sample) was fragmented to 200 bp using a Bioruptor sonicator (Diagenode), and barcode ligation and end repair were achieved using the Ovation Rapid DR Multiplex System 1–96 (NuGEN). Barcoded libraries were combined and sent for SE50 sequencing using an Illumina HiSeq 2500 system at the Genomics Core Facility (Cancer Research UK Cambridge Institute).

Sequence reads were demultiplexed using the Casava pipeline (Illumina) and then aligned to the mouse genome (GRCm38) using Tophat version 2.1.0 (http://ccb.jhu.edu/software/tophat/index.shtml). Differential gene expression was determined using Cufflinks version 2.2.1 (http://cole-trapnell-lab.github.io/cufflinks/).

### Quantitative RT-PCR

Populations of Venus-positive cells (L cells) or Venus-negative cells (non-L cells) of purity greater than 90% were separated from the tissues of GLU-Venus mice using a BD Influx cell sorter running BD FACS Software as previously described (16). Laser alignment was performed using eight-peak rainbow beads (Spherotech), and drop delay was determined using BD Accudrop beads. RNA was extracted from FACS-sorted cells by a microscale RNA isolation kit (Ambion) and reverse transcribed to cDNA according to standard protocols. A first-strand cDNA template was mixed with specific TaqMan primers (Applied Biosystems), water, and PCR master mix (Applied Biosystems), and quantitative RT-PCR was conducted using a 7900HT Fast real-time PCR system (Applied Biosystems). β-Actin was used as the normalization control. The primer/probe pairs used in this study were from Applied Biosystems: *Agtr1*, Mm01957722_s1, and *Mas1*, Mm00434823_s1. All experiments were performed on at least three cDNAs isolated from one mouse each.

### Immunohistochemistry

Tissues were fixed in 4% paraformaldehyde, dehydrated in 15% and 30% sucrose, and frozen in optimal cutting temperature embedding media (CellPath). Cryostat-cut sections (6–10 μm) were mounted directly onto polylysine-covered glass slides (VWR). Slides were incubated for 1 hour in blocking solution containing PBS/0.05% Triton X-100/10% donkey serum and overnight with primary antibodies (goat anti-GLP-1 [sc-7782] and rabbit anti-AT_1_R [sc-579; Santa Cruz Biotechnology Inc]) in blocking solution. Sections were rinsed with blocking solution before being incubated for 1 hour at room temperature with Alexa Fluor 488 (1:300) and Alexa Fluor 555 (1:300) secondary antibodies (Invitrogen) and Hoechst (1:1300) for nuclear staining. Control sections were stained with secondary antibodies alone. Sections were mounted with Prolong Gold (Life Technologies) before being imaged by confocal microscopy (Leica TCS SP8).

### Ussing chamber recordings

The most distal part of the colon (∼1.25 cm) was cut open longitudinally and rinsed in Ringer's solution. Serosa and most of the outer muscular layer were removed by fine forceps. The tissue was mounted in an Ussing chamber (EM-LVSYS-4 system with P2400 chambers and P2404 sliders; all from Physiologic Instruments). Only one preparation from each animal was used. The active epithelial surface was 0.25 cm^2^. Both parts of the Ussing chambers were filled with 3 mL of Ringer's solution, maintained at 37°C, and continuously bubbled with 5% (vol/vol) CO_2_/95% (vol/vol) O_2_. The transepithelial potential difference was clamped to 0 mV using a DVC 1000 amplifier (WPI), and the resulting short circuit current was recorded through Ag-AgCl electrodes and 3 mol/L KCl agarose bridges. The recordings were collected and stored using a Digidata 1440A acquisition system and AxoScope 10.4 software (both from Molecular Devices). The transepithelial resistance and short circuit current (Isc) were allowed to stabilize for at least 30 minutes before the application of drugs. During this period, transepithelial resistance was assessed by measuring current changes in response to 2-mV pulses lasting 2.5 seconds, applied every 100 seconds. After stabilization of the electrical parameters, the following drugs were applied: 5 μmol/L amiloride, 1 μmol/L candesartan, 1 μmol/L BIBP 3226, and 1 μmol/L Ang II. Forskolin (10 μmol/L) was applied bilaterally at the end of each experiment to confirm the responsiveness/viability of the tissue. Because Ang II triggered a sustained depression in Isc in all tissue preparations tested but a short-lived increase (1–3 min duration) in only approximately half the preparations, the difference between the mean Isc 2–5 minutes immediately preceding, and the mean Isc during 30 minutes after Ang II application was used to combine data from different preparations.

### Statistics

Results are expressed as mean ± SD unless otherwise indicated. Statistical analysis was performed using GraphPad Prism 5.01. For GLP-1 and PYY secretion data, a one-way ANOVA with post hoc Dunnett's or Bonferroni tests were performed on log-transformed secretion data because these data were heteroscedastic. For Ussing chamber recordings, a one-way ANOVA with post hoc Dunnett's test was performed on nontransformed Isc data normalized for a surface area of 1 cm^2^. For quantitative RT-PCR (qRT-PCR), a one-way ANOVA with post hoc Bonferroni analysis was done on nontransformed δcycle threshold (Ct) data. Statistical significance for Ca^2+^ imaging data was assessed by a Student's *t* test.

## Results

### AT_1_ receptor expression in mouse and human colonic L cells

**Figure 1.**
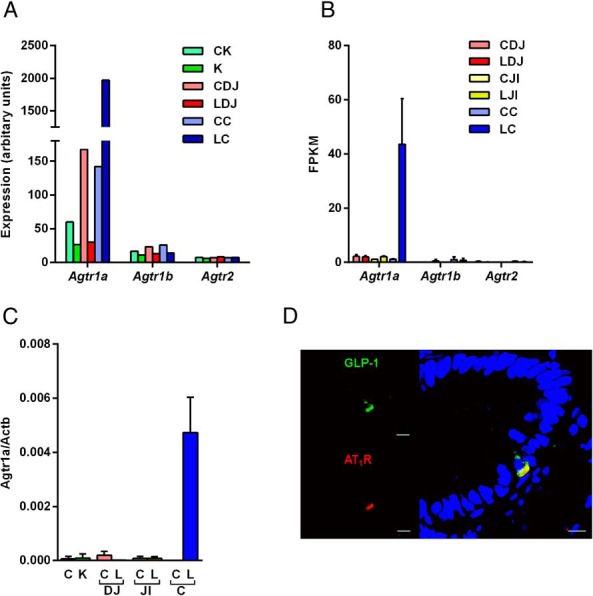
Angiotensin II type 1 receptor (AT1R) is highly and exclusively present in colonic L cells. Gene expression of *Agtr1a*, *Agtr1b*, and *Agtr2* was examined by microarray analysis from FACS-sorted mouse K cells (K), upper small intestinal (duodenal/jejunal) L cells (LDJ), and colonic L cells (LC), together with corresponding nonfluorescent control cells collected in parallel (CK, CDJ, CC, respectively) (A) and by RNA sequencing on FACS-sorted L cells and controls from mouse duodenum/jejunum (LDJ, CDJ), jejunum/ileum (LJI, CJI), and colon (LC, CC) (B). C, *Agtr1a* expression was validated by qRT-PCR in mouse K, L, and control cells. Data are presented as the geometric mean ± upper SEM of the 2δCt data (n ≥ 3 each). Comparisons between L cells and controls were assessed on nontransformed δCt data using a one-way ANOVA and post hoc Bonferroni analysis. ***, *P* < .001. D, Representative photomicrograph demonstrating colocalization of GLP-1 (green) and AT1R (red) in 4% paraformaldehyde-fixed human colon tissue section. Nuclei were visualized with Hoechst staining (blue). Scale bar, 10 μm.

Ang II interacts with two seven-transmembrane G protein-coupled receptors, AT_1_ and angiotensin type 2. Whereas rodents possess two AT_1_ receptor isoforms, AT_1A_ and AT_1B_ (encoded by *Agtr1a* and *Agtr1b*, respectively) (20), humans have only one type 1 receptor gene. Microarray analysis was performed to compare the expression of *Agtr1a*, *Agtr1b*, and *Agtr2* in primary murine glucose-dependent insulinotropic polypeptide secreting K cells as well as L cells from the duodenum/jejunum (top 10 cm of the small intestine) or the colon. As shown in Figure 1A, *Agtr1a* expression was approximately 100-fold higher in colonic (LC) than upper small intestine (SI) L cells (LDJ) and 14-fold enriched in colonic L cells (LC) over non-L cells (CC). *Agtr1b* and *Agtr2* were poorly expressed in all cell populations examined (Figure 1A). RNA sequencing confirmed the high selective expression of *Agtr1a* in colonic L cells (LC, Figure 1B). Microarray and RNA-sequencing results were also validated by quantitative PCR, performed on cDNA prepared from in dependently FACS-sorted L and non-L cells from the upper SI (duodenum/jejunum, LDJ), the lower SI (jejunum/ileum, LJI), and colon (LC) as well as K cells and non-K cells. By quantitative PCR, *Agtr1a* was highly enriched in colonic L cells (LC) over colonic control cells (CC) and was found at much lower levels in small intestinal epithelial control (CDJ, CJI) and L cells (LDJ, LJI) and K and non-K cells (CK) (Figure 1C).

In human colon tissue sections, AT_1_ immunopositive cells were found scattered through the epithelium and costained with antibodies against GLP-1(Figure 1D). No visible staining for AT_1_ was detected in GLP-1-negative cells of the epithelial layer. Some cells in the lamina propria showed AT_1_ reactivity, but their identity was not further investigated (data not shown).

### Ang II-stimulated GLP-1 and PYY secretion from mouse and human colon cultures

**Figure 2.**
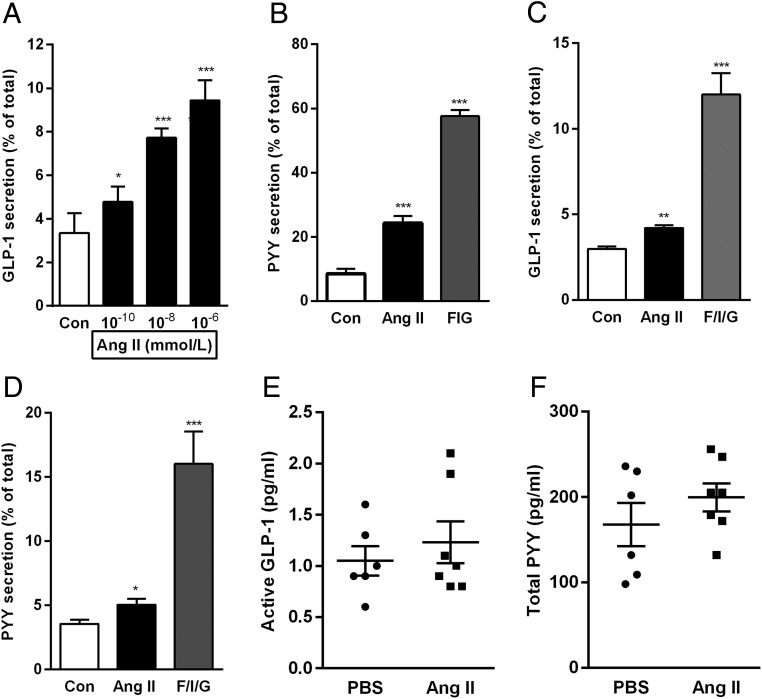
Angi II stimulates GLP-1 and PYY secretion from mouse and human colon cultures. A, GLP-1 secretion was measured from mouse mixed colon cultures incubated for 2 hours in saline solution alone (control [Con]) or containing increasing concentrations of Ang II. B, PYY secretion was measured from mixed cultures incubated with Ang II (10 nmol/L) or forskolin (10 μmol/L) plus 3-isobutyl-1-methylxanthine (10 μmol/L) plus glucose (10 mmol/L) (F/I/G). GLP-1 and PYY secretion is expressed as a percentage of total hormone content in each well. Similarly, GLP-1 (C) and PYY (D) secretion was measured from human colon cultures incubated with Ang II (10 nmol/L) or F/I/G. Results are shown as the mean ± SEM of 12 (A), 13–14 (B), 11–14 (C), 11–15 (D) wells, with three or four wells originating from a single mouse or human tissue sample. *, *P* < .05, **, *P* < .01, ***, *P* < .001 compared with controls using a one-way ANOVA followed by post hoc Bonferroni analysis on log10-transformed data. Active GLP-1 (E) and total PYY (F) levels were measured in plasma of mice that received a single ip injection of either Ang II (100 μg/kg) or PBS (vehicle). Mean ± SEM from six to seven mice per group are depicted.

The functional relevance of the high *Agtr1a* expression in mouse colonic L cells was investigated by performing hormone secretion experiments from primary murine colonic cultures. Cells were incubated for 2 hours with Ang II (10^−10^, 10^−8^, and 10^−6^ mol/L) or with a positive control containing a combination of forskolin (10 μmol/L), 3-isobutyl-1-methylxanthine (10 μmol/L), and glucose (10 mmol/L). Ang II stimulated GLP-1 secretion at all concentrations tested. The highest concentration, 10^−6^ mol/L, increased GLP-1 secretion from 3% to 9.5% of the total GLP-1 content (Figure 2A). Secretion of PYY, which is coreleased from colonic L cells, was examined with a single concentration of Ang II (10^−8^ mol/L) and increased from 8.5% to 24% of the total PYY content (Figure 2B). Consistent with the localization of AT_1_ in human colonic L cells, Ang II (10^−6^ mol/L) also enhanced GLP-1 and PYY secretion by approximately 1.4-fold each (Figure 2, C and D) in human colonic crypt cultures. In mice, acute ip injection of Ang II (100 μg/kg) did not, however, significantly increase plasma GLP-1 or PYY concentrations (Figure 2, E and F).

### GLP-1 and PYY secretion is mediated by AT_1_ receptor

**Figure 3.**
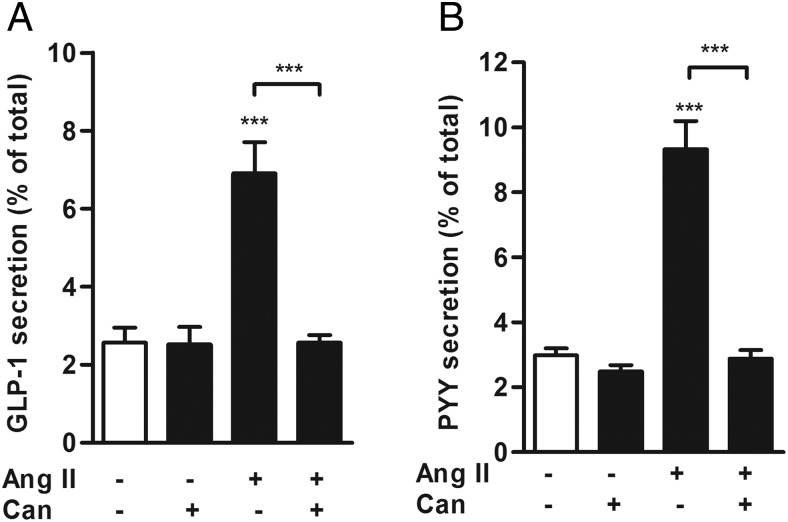
Antagonism of AT_1_ receptor reduces GLP-1 and PYY secretion from mouse colon cultures. GLP-1 (A) and PYY (B) secretion was measured from colon cultures treated with Ang II (10 nmol/L) in the presence or absence of Candesartan cilexetil (Can; 1 μmol/L), a selective AT_1_ receptor antagonist. Where applicable, wells were pretreated with Can 30 minutes before the administration of Ang II. GLP-1 and PYY secretion is expressed as a percentage of total content. Results are shown as the mean ± SEM (n = 9–12 wells, with three or four wells originating from a single mouse. ***, *P* < .001 compared with controls or as indicated using a one-way ANOVA followed by post hoc Dunnett's test or Bonferroni analysis on log10-transformed data.

To investigate whether other receptors for Ang II or its metabolites play a role in Ang II-stimulated hormone secretion from the colon, we performed secretion experiments in the presence of Candesartan cilexetil, a prodrug used to treat hypertension, which is converted to the selective AT_1_ inhibitor Candesartan by the intestinal wall esterases (21). Candesartan (10^−7^ mol/L) had no effect on basal GLP-1 secretion but abolished Ang II-triggered GLP-1 release from mouse colonic cultures (Figure 3A). Ang II-triggered PYY secretion was also blocked by cotreatment with Candesartan (Figure 3B), thereby establishing the role of AT_1_ in mediating Ang II-stimulated GLP-1 and PYY secretion.

### Ang II induced intracellular calcium responses in colonic L cells

**Figure 4.**
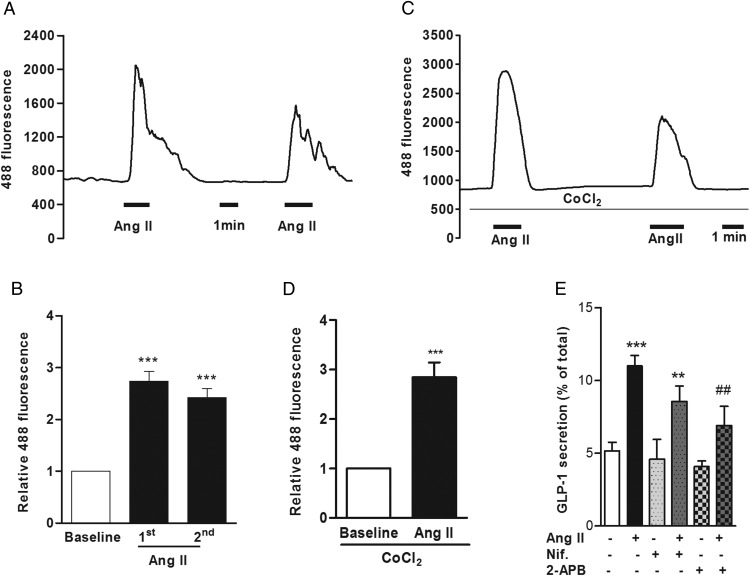
Ang II elevates intracellular calcium responses in colonic L cells. A, A representative trace showing calcium response to Ang II (10 nmol/L) in an L cell from a mixed colon culture imaged by GCaMP3 fluorescence. B, Mean normalized GCaMP3 fluorescence changes in L cells exposed to two successive applications of Ang II, recorded as in panel A (n = 6 cells and results are the mean ± SEM). ***, *P* < .001 compared with baseline by a one-sample Student's *t* test. C, A representative trace showing calcium response to Ang II (10 nmol/L) in the presence of cobalt chloride (CoCl2; 5 mmol/L) to block voltage-gated calcium channels and mean GCaMP3 fluorescence changes in L cells (D) in response to Ang II (10 nmol/L) in the presence of CoCl_2_ (n = 12 cells). Results are shown as the mean ± SEM. ***, *P* < .001 compared with baseline by a one-sample Student's *t* test. E, GLP-1 secretion from mouse mixed colon cultures stimulated with Ang II (10 nmol/L) in the presence or absence of nifedipine (Nif; 10 μmol/L) or 2-APB (100 μmol/L). Where applicable, wells were pretreated with nifedipine or 2-APB 30 minutes before the administration of Ang II. GLP-1 secretion is expressed as a percentage of total content. Results are shown as the mean ± SEM (n = 10–12 wells with three or four wells originating from a single mouse). ***, *P* < .001 compared with controls; ##, *P* < .01 compared with Ang II alone as indicated, using a one-way ANOVA followed by post hoc Dunnett's test or Bonferroni analysis on log10-transformed data.

Previous studies have revealed that AT_1_ receptor activation recruits phospholipase C and stimulates the hydrolysis of phosphatidylinositol-4,5-bisphosphate to diacylglycerol and inositol-1,4,5-trisphosphate (IP3), promoting calcium release from internal stores (22, 23). Depending on the cell or tissue type, it was also reported that Ang II inhibits adenylate cyclase and lowers intracellular cAMP levels (24, 25). To elucidate the mechanistic pathway involved in Ang II-triggered GLP-1 and PYY release, we monitored the changes in intracellular calcium in primary colonic L cells identified in cultures from GLU-Cre/ROSA26-GCaMP3 mice during Ang II application. As shown in Figure 4A, Ang II triggered a rapid increase in L-cell GCaMP3 fluorescence, indicative of an increase in the intracellular calcium concentration. Responses peaked shortly after Ang II addition, were rapidly reversible, and were reproducible on a second application of Ang II (Figure 4, A and B).

Intracellular calcium can be increased either by opening of plasma membrane calcium channels or by release from intracellular calcium stores. To establish whether the Ang II-dependent cytoplasmic calcium rise was due to calcium release from intracellular endoplasmic reticulum stores or the opening of plasma membrane voltage-gated calcium channels, calcium imaging experiments were performed in the presence of cobalt chloride (CoCl_2_), a general voltage-gated calcium channel blocker that impairs L-cell calcium responses to depolarizing stimuli such as KCl (26). Cytoplasmic calcium responses to Ang II were still observed in the presence of CoCl_2_ (5 mmol/L) (Figure 4, C and D), suggesting they do not depend on voltage-gated calcium channels. This is consistent with the reported Gq-coupled nature of AT_1_ (27). Further corroborating the results obtained with calcium imaging experiments, the L-type voltage-gated calcium channel blocker nifedipine (10 μmol/L) did not significantly inhibit Ang II-stimulated GLP-1 secretion (Figure 4E), but GLP-1 responses to Ang II were blocked by 2-aminoethoxydiphenylborate (2-APB, 100 μmol/L), an inhibitor of IP3 receptors (Figure 4E).

### Nonclassical RAS and L cells

**Figure 5.**
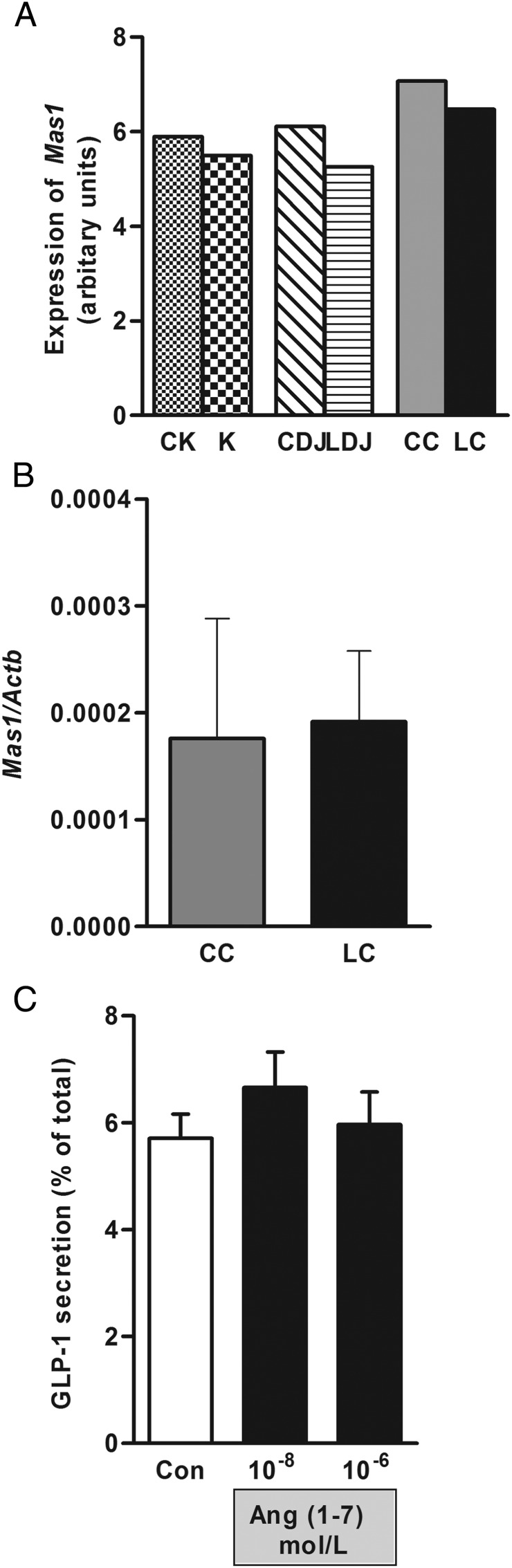
Angiotensin (1–7) and the Mas1 receptor are not involved in GLP-1 secretion. Mas1 receptor expression was analyzed by microarray analysis on FACS-sorted K and L cells from duodenum/jejunum (LDJ) and colon (LC) and respective control cells (CDJ, CC) (A) and by qRT-PCR on colonic L and control cells (B). qRT-PCR data are presented as the geometric mean ± upper SEM of the 2δCt data (n ≥ 3 each). C, GLP-1 secretion was measured from mouse colon cultures in the presence of two concentrations of angiotensin (1–7). GLP-1 secretion is expressed as a percentage of total content. Results are shown as the mean ± SEM (n = 9–12 wells, with three or four wells originating from a single mouse). Statistics were performed using a one-way ANOVA followed by post hoc Dunnett's on log 10-transformed data.

Whereas the classical RAS (ACE-Ang II-AT_1_) promotes actions to maintain BP, a nonclassical RAS, consisting of ACE2-Ang (1–7)-Mas1 receptor has opposing effects (28). Angiotensin (1–7) is generated by the cleavage of an amino acid from the carboxy-terminus of Ang II by an ACE homologue, ACE2, and mediates vasodilatory/diuretic actions through the AT_7_/Mas1 receptor (29). Microarray (Figure 5A) and RNA-sequencing analysis (data not shown) for *Mas1* receptor expression were performed on K and L cells from mouse upper SI (LDJ) and colon (LC) and their respective control cells (CK, CDJ, CC). *Mas1* expression was very low or undetectable in all cell populations examined. This was confirmed by quantitative PCR (Figure 5B). Consistent with these findings, application of Ang (1–7) to primary murine colonic crypt cultures had no significant effect on GLP-1 release (Figure 5C).

### Antisecretory effect of Ang II in mouse colon

**Figure 6.**
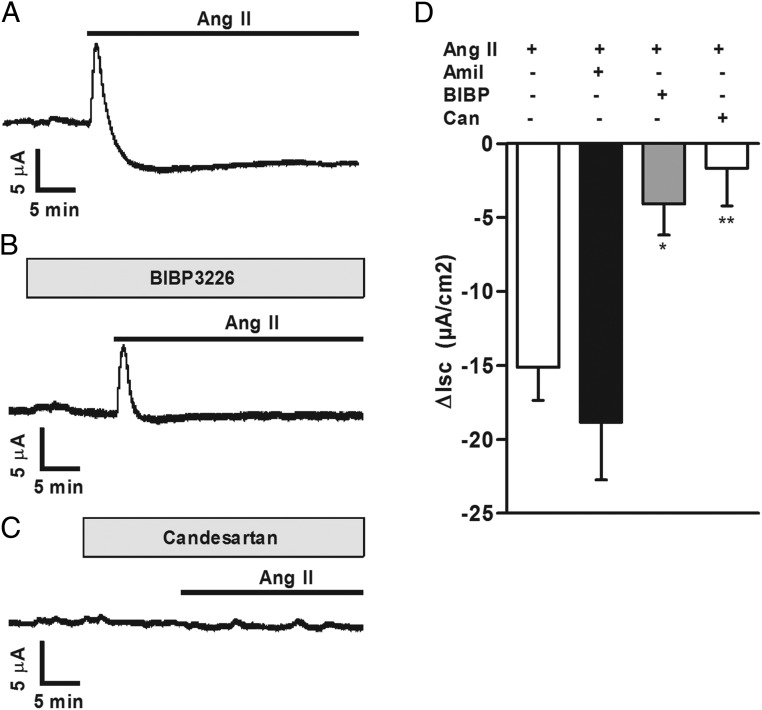
Ang II-induced effect on short circuit current in mouse distal colon. A, Example traces showing changes in short circuit current recordings (Isc) from mouse distal colon mounted in Ussing chambers after basolateral application of Ang II (1 μmol/L). B, Isc changes from colon tissue as in panel A but in the additional presence of basolateral NPY1R antagonist BIBP3226 (1 μmol/L). C, Isc changes from colon tissue as in panel A but in the additional presence of bilateral Ang II type 1 receptor antagonist Candesartan (Can; 1 μmol/L). D, Mean changes in Isc, recorded as in panels A–C, after application of Ang II alone or in the presence of amiloride (Amil; 5 μmol/L), BIBP3226 (BIBP), or Can. δIsc was calculated as the difference between the means of short circuit currents from the 2- to 5-minute period before and 30-minute period after the application of Ang II. Data are the mean ± SEM from four to five tissue preparations for each condition, normalized for a surface area of 1 cm^2^. *, *P* < .05, **, *P* < .01 compared with Ang II application alone using a one-way ANOVA followed by post hoc Bonferroni analysis on nontransformed data.

Given the well-known inhibitory effect of PYY on intestinal anion and water secretion (4, 5, 30–32), we used Ussing chambers to study the functional relevance of Ang II in mouse colon. In all tissue preparations tested, basolateral addition of Ang II (10^−6^ mol/L) caused a sustained depression in Isc (of mean 15.1 μA/cm^2^) lasting for at least 35 minutes (Figure 6A). In three of five preparations, we also observed a transient increase in Isc, with a peak increase of 40.6 ± 10.1 μA/cm^2^ (Figure 6A), but this was absent in the other two preparations (not shown). Pretreatment with apically added amiloride (5 μmol/L) alone decreased Isc, which came to a new plateau 9.6 ± 9.1 μA/cm^2^ lower than the Isc before amiloride addition. Subsequent application of Ang II 10–12 minutes after amiloride pretreatment caused further Isc depression, which was not different from the response caused by Ang II without any pretreatment (Figure 6D). These results suggest that the Ang II-related Isc decrease was due to inhibition of electrogenic anion secretion and did not involve epithelial sodium channel-dependent sodium absorption. Pretreatment with basolaterally added BIBP3226 (BIBP; a specific neuropeptide Y-1 receptor [NPY1R] antagonist) caused an increase of Isc, with the new plateau being 1.8 ± 2.7 μA/cm^2^ higher than before the BIBP addition. The inhibitory Isc response to Ang II applied 10–12 minutes after BIBP was significantly impaired, confirming a role of the PYY receptor NPY1R in Ang II-mediated changes of colonic transepithelial ion movement (Figure 6, B and D). Candesartan (10^−6^ mol/L bilaterally) reduced the basal Isc by1.9 ± 2.8 μA/cm^2^ and abolished any subsequent responses to Ang II application, confirming the role of AT_1_ in Ang II-stimulated Isc changes (Figure 6, C and D).

The above-mentioned initial short-lived (1–3 min) Isc increase after Ang II addition was observed in two of four preparations pretreated with amiloride, two of four preparations pretreated with BIBP, and none of four preparations pretreated with Candesartan. When considering all preparations together, there was no significant difference in the early peak magnitude between the groups (data not shown).

## Discussion

Digestion and absorption of nutrients from the intestine depends on sufficient availability of water in the lumen. Indeed, in addition to the average ingested fluid volume of approximately 2.5 L/d in humans, it has been estimated that 5–10 L of water are secreted into the gut lumen and reabsorbed to aid intestinal processes (33), necessitating a close link between the gut and systems regulating body fluid and electrolyte homeostasis. Here we identified AT_1_ in colonic L cells and demonstrated that its activation by Ang II triggered GLP-1 and PYY secretion and downstream PYY-dependent inhibition of anion secretion. This offers a potential explanation for previous reports that colonic fluid secretion is regulated by the renin angiotensin system (15).

The effect of Ang II on intestinal water and ion absorption has been studied extensively in the rat. At low physiological concentrations, Ang II stimulates water absorption in the jejunum and colon, although higher doses were also reported to inhibit absorption (15). In the jejunum the proabsorptive effect of Ang II was linked to the activation of noradrenergic nerve endings, based on the sensitivity of the response to α-adrenergic antagonists (34). Early investigations concluded that the proabsorptive effects of Ang II are predominantly mediated by electroneutral mechanisms (13), but experiments on rat descending colon mounted in Ussing chambers revealed a reduction of Isc over a wide Ang II concentration range (10^−9^ to 10^−5^ mol/L) (14). This was sensitive to the chloride channel blocker, diphenylamine-2-carboxylate, but not to amiloride, suggesting that the action of Ang II on Isc is mediated through inhibition of anion secretion rather than stimulation of electrogenic sodium absorption (14).

Our observed decrease in Isc in the colon is in agreement with these results and is clearly mediated via AT_1_ because it was sensitive to Candesartan. In about half the preparations, we also observed a transient increase in Isc; this might also be downstream of AT_1_ because it was never observed in the presence of candesartan, but in contrast to the sustained Isc reduction, it was not affected by the Y1 receptor antagonist BIBP3226. Our finding that the sustained reduction in Isc by Ang II was sensitive to BIBP3226 and insensitive to amiloride suggests that this effect lies downstream of PYY secretion. PYY, in addition to slowing gastric emptying and reducing hunger, is well recognized as an inhibitor of anion and electrolyte secretion (35), exerting its inhibitory action mainly via Y1 receptors on enterocytes and to some extent by Y2 receptors on enteric neurons (31). Activation of the Gi/Go coupled Y1 receptor lowers intracellular enterocyte cAMP levels, subsequently inhibiting cystic fibrosis transmembrane conductance regulator channels and thereby reduces anion secretion into the gut lumen (4).

Previous studies have shown that AT_1_ is the predominant Ang II receptor in the muscularis of rat ileum and colon (7), submucosal plexus in guinea pig distal colon (36), vessel walls, myofibroblasts, and macrophages in the lamina propria, crypt bases, and surface epithelium in the human colon (9) as well as a subset of human jejunal cells resembling enteroendocrine cells (37). Our data contrast with the previously reported detection of AT_1_ in jejunal enterocytes (37) because we found only very low mRNA expression in the non-L cell population of the mouse small intestine, which would be dominated by enterocytes. Although this might reflect species differences, we also observed clear AT_1_ staining in human colonic L cells but not enterocytes. The fact that we were able to block the sustained drop of short circuit current observed in Ussing chamber-mounted colonic tissue in response to Ang II with the Y1R blocker BIBP3226 is consistent with the observed restriction of AT_1_ to L cells in the murine colon and an important role of L cells in the secretory responses of the colon to Ang II.

Although Ang II could in principle also exert some of its effects through other receptors, we were unable to demonstrate a role of other angiotensin receptors in L cells. mRNAs encoding both angiotensin type 2, which has a similar affinity for Ang II as AT_1_, and the *Mas1* receptor were expressed only at very low levels, barely detectable by RT-PCR. Angiotensin (1–7), the ligand for MAS1, had no effect in GLP-1 secretion. The effects on gut hormone secretion of other angiotensin-derived peptides such as angiotensin III and angiotensin IV have not been studied, and a possible function cannot be ruled out. However, Candesartan, a specific antagonist for AT_1_, completely abolished Ang II-triggered GLP-1 and PYY secretion, emphasizing the predominant role of this receptor for Ang II-stimulated gut hormone release. In keeping with the known Gq coupling of AT_1_ in heterologous expression systems, we observed Ang II-triggered Ca^2+^ responses that were maintained in the presence of extracellular Co^2+^, a treatment that eliminates Ca^2+^ rises downstream of voltage-gated Ca^2+^ channels in L cells (38). Consistent with these results, nifedipine, which blocks L-type voltage gated Ca^2+^ channels and inhibits GLP-1 secretion from L cells (39), had no significant effect on Ang II-stimulated secretion. Sensitivity of the secretory response in L cells to 2-APB, an inhibitor of IP_3_ receptors, is consistent with the recruitment of endoplasmic reticulum stores, although we cannot exclude additional contributions from plasma membrane channels such as transient receptor potential channels, a number of which are expressed in L cells (38) sensitive to 2-APB.

### Physiological relevance

Our results suggest that physiological activation of the renin angiotensin system will be accompanied by increased colonic GLP-1 and PYY secretion and are in keeping with our previous report that colonic L cells are also activated by AVP. Whereas PYY likely exerts local actions on fluid secretion, it is not known whether Ang II-dependent stimulation of colonic L cells would be sufficient to elevate circulating GLP-1 and PYY levels and trigger anorexigenic and insulinotropic responses. Chronic infusion of Ang II at a rate of 1.5 μg/(kg·min) has been shown to reduce food intake in C57B6 mice in a Candesartan-sensitive manner (40). Although no changes in intestinal hormone mRNA expression were observed, circulating hormone levels were not reported. However, we failed to detect significant changes in plasma GLP-1 or PYY in response to ip Ang II injection (100 μg/kg), a supraphysiological dose chosen approximately 3- to 20-fold in excess of doses previously reported to affect taste behavior (41) and BP (42) in mice. Whereas this might support the view that the well-documented anorexic effects of Ang II are downstream of direct action in the central nervous system (43), it is also well known that anorexic effects of enteroendocrine hormones are at least in part mediated via afferent neuronal fibers, which could be stimulated by local elevations of gut hormones insufficient to raise plasma levels (2).

Despite the enrichment of AT_1_ receptors in colonic L cells and the finding that AT_1_ receptor activation triggered GLP-1 and PYY release, these receptors would not seem a promising target for drug discovery in the field of L-cell secretagogues. Although it has been proposed that local AT_1_ agonism in the jejunum might beneficially reduce sodium-dependent glucose transporter-1-mediated glucose absorption (37, 44), the potential benefits of targeting intestinal AT_1_ receptors do not weigh favorably against the evident clinical cardiovascular benefits of ACE inhibitors and angiotensin receptor blockers. Nevertheless, the finding that AVP and angiotensin receptors are highly enriched in colonic L cells raises the concept of an important cross talk between colonic enteroendocrine cells and fluid balance regulatory pathways and raises interesting questions about the physiological control and functional roles of colonic hormones.

## Appendix

**Table 1.** Antibody Table

Peptide/Protein Target	Antigen Sequence (if Known)	Name of Antibody	Manufacturer, Catalog Number, and/or Name of Individual Providing the Antibody	Species Raised (Monoclonal or Polyclonal)	Dilution Used
GLP-1	Not available	GLP-1 antibody (C-17)	Santa Cruz Biotechnology, sc-7782	Goat, polyclonal	1:100
AT_1_ receptor	Not available	AT_1_ antibody (306)	Santa Cruz Biotechnology, sc-579	Rabbit, polyclonal	1:100
